# Pathways of exposure to *Vibrio Cholerae* in an urban informal settlement in Nairobi, Kenya

**DOI:** 10.1371/journal.pgph.0002880

**Published:** 2024-08-20

**Authors:** Kelvin Kering, Yuke Wang, Cecilia Mbae, Michael Mugo, Beatrice Ongadi, Georgina Odityo, Peter Muturi, Habib Yakubu, Pengbo Liu, Sarah Durry, Aniruddha Deshpande, Wondwossen Gebreyes, Christine Moe, Samuel Kariuki

**Affiliations:** 1 Centre for Microbiology Research, Kenya Medical Research Institute, Nairobi, Kenya; 2 Center for Global Safe Water, Sanitation, and Hygiene, Hubert Department of Global Health, Rollins School of Public Health, Emory University, Atlanta, Georgia, United States of America; 3 Department of Epidemiology, Rollins School of Public Health, Emory University, Atlanta, Georgia, United States of America; 4 Global One Health initiative (GOHi), The Ohio State University, Columbus, Ohio, United States of America; 5 Veterinary Preventive Medicine, The Ohio State University, Columbus, Ohio, United States of America; Kansas State University, UNITED STATES OF AMERICA

## Abstract

Cholera is a diarrhoeal disease caused by *Vibrio cholerae* (*V*. *cholerae*) bacterium, with strains belonging to serogroups 01 and 0139 causing a huge proportion of the disease. *V*. *cholerae* can contaminate drinking water sources and food through poor sanitation and hygiene. This study aimed to identify environmental routes of exposure to *V*. *cholerae* within Mukuru informal settlement in Nairobi. We collected nine types of environmental samples (drinking water, flood water, open drains, surface water, shaved ice, raw produce, street food, soil, and public latrine swabs) over 12 months. All samples were analysed for *V*. *cholerae* by culture and qPCR, then qPCR-positive samples were quantified using a *V*. *cholerae* DNA standard. Data about the frequency of contact with the environment was collected using behavioural surveys. Of the 803 samples collected, 28.5% were positive for *V*. *cholerae* by qPCR. However, none were positive for *V*. *cholerae* by culture. *V*. *cholerae* genes were detected in majority of the environmental water samples (79.3%), including open drains, flood water, and surface water, but were only detected in small proportions of other sample types. Vibrio-positive environmental water samples had higher mean *V*. *cholerae* concentrations [2490–3469 genome copies (gc) per millilitre (mL)] compared to drinking water samples (25.6 gc/mL). Combined with the behavioural data, exposure assessment showed that contact with surface water had the highest contribution to the total *V*. *cholerae* exposure among children while ingestion of municipal drinking water and street food and contact with surface water made substantial contributions to the total *V*. *cholerae* exposure for adults. Detection of *V*. *cholerae* in street food and drinking water indicates possible risk of exposure to toxigenic *V*. *cholerae* in this community. Exposure to *V*. *cholerae* through multiple pathways highlights the need to improve water and sanitation infrastructure, strengthen food hygiene practices, and roll out cholera vaccination.

## Introduction

Cholera is an acute infectious diarrhoeal disease caused by toxigenic *Vibrio cholerae* (*V*. *cholerae*), a comma-shaped, gram-negative bacterial pathogen [1, 2] transmitted to humans through the consumption of fecal-contaminated food and water. Toxigenic strains belonging to serogroups 01 and 0139 are responsible for majority of the cholera cases reported [3, 4]. *V*. *cholerae* serogroup 01 biotype El Tor is endemic in Africa after multiple introductions from Asia and is responsible for most of the epidemics reported in sub-Saharan Africa [5, 6]. Whereas serogroups 01 and 0139 are responsible for pandemic cholera outbreaks, non-01/0139 *V*. *cholerae* (NOVC) serogroups cause mild gastroenteritis to violent diarrhoea and localized outbreaks [7–9].

It is estimated that approximately 2.9 million cases of cholera occur annually, resulting in 95,000 deaths globally, with low- and middle-income countries bearing the biggest burden [10]. Of the cholera cases reported to the World Health Organization (WHO) between 1996 and 2019 [11, 12] the majority were reported from sub-Saharan Africa. In Kenya, cholera was first reported in 1971, with several outbreaks being reported every five to seven years [13, 14]. Cholera cases are often recorded in refugee camps [15, 16] and Nairobi’s urban informal settlements [17] with case fatality rates (CFR) greater than 2.5%. For instance, in 2016, cholera cases were reported in 22 of Kenya’s 47 counties [16] with an average CFR of 1.6 [14], while in 2019, 5208 cases were recorded, with a CFR of 2.7% [14]. The high CFR observed in Kenya is concerning and highlights the need to map cholera hotspots, identify risk factors, and implement evidence-based integrated intervention strategies.

*V*. *cholerae* is pervasive in aquatic environments and thrives as free-living cells in brackish and freshwater or biofilms on zooplankton and phytoplankton [2, 18]. However, in unfavourable environmental conditions and limited nutrition, *V*. *cholerae* cells enter a dormant state referred to as viable but non-culturable (VBNC) or conditionally viable environmental cells (CVEC) of *V*. *cholerae* [19–21]. During the VBNC and CVEC state, *V*. *cholerae* cells retain virulence, and their pathogenic potential [22] and can revert to a culturable state or active form after passing through human or animal intestines. From aquatic environments *V*. *cholerae* can spread into drinking water sources and food through fecal-contaminated runoff from flooding and/or wastewater [23], thereby exposing the human population. The presence and persistence of *V*. *cholerae* in the environment (freshwater, wastewater, rivers) [24–26] enables exposure assessment using data on human behaviour interactions with the environment and the levels of *V*. *cholerae* in these environments.

Impoverished populations, especially in informal settlements [13], refugee camps, and areas afflicted by natural calamities and conflicts [27] are disproportionately affected by cholera due to poor water, sanitation, and hygiene (WASH), limited access to healthcare, and weak healthcare systems. Due to rapid population growth and unplanned residential developments, it is estimated that as of 2023 about 60% of Kenya’s population reside in informal settlements and slums [28, 29], some of which are located within Kenya’s capital, Nairobi. One of the informal settlements in Nairobi, Mukuru Slums, is characterized by overcrowding, poor solid-waste management, inadequate WASH infrastructure and limited access to healthcare. While there have been previous reports of cholera outbreaks within the Mukuru informal settlements, the probable pathways of exposure have not been identified.

SaniPath is an exposure assessment approach that identifies and characterizes the pathways of exposure to fecal contamination and enables the prioritization and design of WASH interventions [30, 31]. The SaniPath Assessment Tool [32] provides guidance to design and conduct multi-pathway exposure through key informant interviews, transect walks, behaviour surveys, environmental sampling, laboratory analysis, and statistical modelling. This study extended the SaniPath approach for assessing exposure to fecal contamination to exposure specifically to *V*. *cholerae*. Our goal was to understand the transmission of *V*. *cholerae* by examining plausible pathways of exposure to *V*. *cholerae* and examining spatial and temporal variation in *V*. *cholerae* detection and concentration in different environmental samples. The results from this study will guide the development of evidence-based integrated prevention and control strategies for this cholera-endemic setting.

## Materials and methods

### Study site

The study was conducted in the Mukuru informal settlement, which is located 20 km east of Nairobi in Kenya. This informal settlement is divided into eight villages and this study was conducted in two villages (Mukuru Kwa Njenga and Mukuru Kwa Reuben), hereafter referred to as neighbourhoods. The neighbourhoods are further divided into zones. Based on local hospital records (from the Mukuru Kwa Reuben Health Centre, Mukuru Health Centre, and Medical Missionaries of Mary) and community health volunteers (CHVs) reports of previous cholera cases, the study was conducted in five zones identified as cholera hotspots (zones that had reported cholera cases in the most recent outbreak, 2018) [33]. Three zones (Gatoto, Mombasa, Feed the Children-FTC) within Mukuru kwa Reuben and two zones (Wapewape and Pipeline) within Mukuru kwa Njenga neighbourhood were selected as study sites.

### Identification of exposure pathways

Transect walks (walking across neighbourhoods/villages while collecting information such as Global Positioning System (GPS) coordinates and environmental features pertinent to fecal contamination) [34] and information from CHVs were used to identify plausible exposure pathways to*V*. *cholerae*. The transect walks were conducted as previously described [32, 35]. Data from the transect walks and information provided by the CHVs revealed the diverse pathways of fecal contamination relevant to the Mukuru informal settlement and the most appropriate environmental sampling sites. The sampling sites were selected in areas where the residents often interacted with those pathways, including the children’s playgrounds, the most frequently used public sanitation facilities, common public water sources, and the most commonly consumed produce from markets and street foods from neighbourhood street vendors.

### Behaviour survey and environmental sample collection

Between November 2020 and April 2022, we conducted 808 household surveys from randomly selected households, 16 community surveys (average of 18 participants per survey), and 16 school surveys (20 participants per survey) in the study neighbourhoods using the SaniPath Tool [32, 36]. Respondents to the household surveys were adults residing in the selected households who were involved in the management of water within the household and familiar with children’s activities. The community and school surveys were conducted separately for male and female participants to help reduce potential reporting biases that could be influenced by gender roles in the community. Participants in the community survey were adults residing within the two study neighbourhoods and with a child aged 5–12 years. The school surveys were conducted in four schools in the two neighbourhoods with school children aged 10–12 years. The surveys collected behaviour information related to the frequency of contact with different compartments of the environment (e.g., contact with open drains, flood water, and surface water, consumption of drinking water, consumption of street food and raw produce, etc.) by adults and children.

In the two neighbourhoods, ten sampling sites (five per neighbourhood) were randomly selected for each sample type. Six sample types (public latrine swabs, soil, surface water, flood water, raw produce, and shaved ice) were collected weekly for 4 months (July–November 2021). The other three sample types (drinking water, open drain, and street food) were collected weekly for one year (July 2021–July 2022). Samples were collected as previously described [35]. Briefly, 1) soil was collected from common children’s playgrounds and public gathering areas; 2) surface water from areas along the rivers where there was frequent human activity or river crossing; 3) public latrines wall surfaces (100 cm^2^) that are frequently touched and door handles were swabbed using two swabs per latrine; 4) flood water was collected from areas with human activity; 5) open drain water was collected from concrete and unlined open drainage channels containing liquid and solid waste, rain, flood water, and wastewater from toilets and household activities in areas where reported human contact was frequent; 6) drinking water was collected from communal municipal water points, boreholes, water vendors, and stored drinking water from randomly selected households where surveys were conducted; 7) street food reported to be commonly consumed by neighbourhood children and adults, 8) produce reported to be commonly consumed raw, and 9) shaved ice purchased from vendors.

Prior to sampling drinking water, chlorine concentration was determined. Drinking water was collected directly from the source into 100 mL Whirl-pak bags containing sodium thiosulphate. Surface water, flood water, and open drain water samples were collected using a sterile metal bucket. The bucket was then placed on the ground to allow the water to settle for five minutes, thereafter the water was poured into 2L Whirl-pak bags. Soil samples were collected at a 45° angle to a depth of 5cm using a sterile spatula. Hard-packed soil samples were gently broken using the spatula before placing them in 100 mL Whirl-pak bags. Street food was collected from vendors according to the quantity normally served to an individual. Raw produce samples included tomato and coriander, and these were collected and placed into 2L Whirl-pak bags. The shaved ice was also collected in quantities typically consumed by an individual and placed in Whirl-pak bags. Public latrine surfaces were swabbed using Puritan environmental sampling swabs pre-moistened with neutralizing buffer and 0.1% peptone water (EnviroMax Plus, Puritan Medical Products Company LLC, Maine, USA). The Whirl-pak bags containing the samples were then placed in cooler boxes containing ice packs and transported to the Microbiology Laboratory at KEMRI and processed within six hours of sample collection.

### Microbiological culture of samples for *Vibrio cholerae*

All samples were initially enriched in Alkaline Peptone Water (APW). For surface water, flood water, open drain water, drinking water, and shaved ice, 5 mL of sample was mixed with 5 mL of APW in 50 mL conical tubes and incubated at 37°C for 18 hours. For raw produce, street food, and latrine swabs, samples were rinsed and then 5 mL of the rinse solution was mixed with 20 mL of APW and incubated. Soil was initially mixed with distilled water and then 5 mL of the soil suspension was mixed with APW and incubated. Shaved ice was allowed to melt at room temperature before it was mixed with APW. The samples were then subcultured onto Thiosulfate Citrate Bile salts Sucrose (TCBS) agar and incubated at 37° C for 18 hours. Suspected *V*. *cholerae* colonies were subjected to both an oxidase test (Thermo Scientific Remel BactiDrop Oxidase) and *V*. *cholerae* 01 polyvalent antisera.

### Sample concentration

The samples were concentrated before DNA extraction. For drinking water (borehole water, water from vendors, and stored drinking water), 100 mL of each sample was filtered through a 0.45 μM membrane filter (Millipore). The membrane filters were then used as the template for DNA extraction. One hundred millilitres of open drain, flood water, or surface water was concentrated using 1% Bovine Serum Albumin (BSA), 12% Polyethylene Glycol (PEG), and 0.9M Sodium Chloride (NaCl). The street foods were initially rinsed with 100 mL of sterile water, after which the homogenized sample was concentrated using 1% BSA, 12% PEG, and 0.9M NaCl as described above. The raw produce was mixed with 100 mL of phosphate-buffered saline Tween (PBST), thereafter the solution was concentrated as described above. For soil, 100 g was mixed with 200 mL of distilled water by shaking vigorously for 30 minutes, and then the sample was allowed to settle at room temperature for two hours. One hundred millilitres of the sample were then concentrated as described previously. Each public latrine swab was mixed with 7 mL of PBST, vortexed briefly, and allowed to incubate at room temperature for 5 minutes and then eluted with PBST. This was done twice for each of the two swabs collected from each public latrine. Approximately 15 mL of the recovered eluate was concentrated using BSA, PEG, and NaCl as described above. The concentrated samples and membrane filters were then stored at 4°C until further processing.

### DNA extraction

The samples were centrifuged at 4200 revolutions per minute for one hour, and the pellet was used for DNA extraction using the QIAamp FAST DNA stool Mini Kit (50) (Qiagen, Hilden, Germany), according to the manufacturer’s instructions. A known *Vibrio cholerae* isolate was used as a positive control, and sterile distilled water was used as the negative control. The extracted DNA was stored at -80°C for further downstream analysis.

### Quantitative polymerase chain reaction (q-PCR) detection of *V*. *cholerae*

The presence of *V*. *cholerae* was determined through the detection of the hemolysin A (*hlyA*) virulence gene by qPCR as described by Huang *et al* [37]. The 25 μL master-mix contained 12.5 μL Bio-Rad iQ Multiplex Powermix, 0.4 μM of each *hlyA* forward and reverse primers, 0.2 μM *hlyA* probe, 5 μL molecular water and 5 μL of DNA template. AMPLIRUN *Vibrio cholerae* DNA control (VIRCELL Microbiologists, MBC118) was used as the positive control, and nuclease-free water was used as the negative control. Quantitative PCR was run on a Magnetic Induction Cycler (MIC) using the following conditions: activation (at 95°C for 15 minutes) and 40 cycles of 95°C for 15 s, 55°C for 40 s and 72°C for 30 s. PCR reactions were run in duplicate wells for each sample. Samples with both Ct values of ≤ 39 and a difference between the duplicate Ct values of < 2 were considered positive.

### Quantification of *V*. *cholerae* in positive samples

*V*. *cholerae* positive samples with two Ct values of ≤ 36 were subjected to quantitative detection of genome copies as previously described [38]. Briefly, commercially purchased AMPLIRUN *Vibrio cholerae* DNA standard (VIRCELL Microbiologists, MBC118) was serially diluted and subjected to qPCR to derive a standard curve. The standard curve was then used to quantify the genome copies in the positive samples. Quantification of genome copies in each sample was estimated by interpolation of the mean Ct value to the standard curve and the dilution factor for each sample type. Absolute equivalent genome copies were expressed as per mL for the water samples, per gram for the raw produce, soil, and street food samples, and per swab for the public latrine samples [38].

### Statistical analysis

We summarized the percent of environmental samples with detected *V*. *cholerae* (positivity) and the mean concentration of *V*. *cholerae* among positive samples by pathway and neighbourhood. Temporal trends of the percent positive samples for each sample type, especially for drinking water, street food, and open drains, were examined. We conducted the SaniPath multi-pathway exposure assessment [32] for *V*. *cholerae* for adults and children. The frequencies of contact with different environmental compartments and the concentrations of *V*. *cholerae* in environmental sample types were modelled using the negative binomial distribution and lognormal distribution, respectively. The parameters of distributions were estimated using Bayesian frameworks by JAGS. Along with the intake volumes of behavioural contacts, exposure by pathway was assessed using Monte Carlo simulations with estimated parameters [36]. The details of the model assumptions and settings (**S1 Text**) was previously described by Raj et al. 2020 [32]. All the analyses were conducted in R version 4.0.1.

### Ethical considerations

Ethical approval to conduct this study was sought and obtained from the Kenya Medical Research Institute review board; Scientific and Ethics Review Unit (**KEMRI/SERU/CMR/P00116/3871**), the National Commission for Science, Technology and Innovation (**NACOSTI/P/20/4566**) and the Nairobi City County (**CMO/NRB/OPR/VOL1-2/2020/32**). All adult participants were requested to participate in the surveys after providing written informed consent. In the case of school children, written informed consent was sought from their parents/guardians and the school administration.

## Results

### *V*. *cholerae* detection by environmental pathway

Based on the transect walks, nine sample types (**Table 1**) were identified as the most probable to be associated with exposure to *V*. *cholerae* within the Mukuru informal settlement. Few water vendors were found in Mukuru Kwa Reuben, therefore, a lesser number of water samples was collected from this location. Of the 803 environmental samples analyzed, none were culture-positive for *V*. *cholerae*. However, 229 (28.5%) samples were positive for the *V*. *cholerae* hemolysin A gene by qPCR detection.

**Table 1:** Sample size and *V*. *cholerae* detection by sample type and neighbourhood.

Percent of Positive (Number of Positive/Number of Samples)			
Sample types	Mukuru Kwa Reuben	Mukuru Kwa Njenga	Total
Drinking water	13.1 (14/107)	11.3 (17/150)	12.1 (31/257)
Flood water	73.3 (11/15)	80.0 (12/15)	76.7 (23/30)
Open drain	73.3 (33/45)	82.6 (38/46)	78.0 (71/91)
Public latrine	40.0 (6/15)	46.7 (7/15)	43.3 (13/30)
Raw produce	20.0 (3/15)	26.7 (4/15)	23.3 (7/30)
Shaved ice	21.4 (3/14)	0 (0/15)	10.3 (3/29)
Soil	26.7 (4/15)	40.0 (6/15)	33.3 (10/30)
Street food	15.0 (21/140)	16.1 (22/137)	15.5 (43/277)
Surface water	93.3 (14/15)	100 (14/14)	96.6 (28/29)
Total	28.6 (109/381)	28.4 (120/422)	28.5 (229/803)

Street food includes french fries, githeri, and mandazi. Drinking water includes municipal water, borehole water, water from vendors, and stored drinking water. Public Latrine samples were swabs of latrine walls and door handles.

In the two study neighbourhoods, surface water had the highest percentage (96.6%) of *V*. *cholerae*-positive samples. For open drain and flood water, 78% and 76.7% of the samples, respectively, were positive for *V*. *cholerae* (**Table 1**). The sample types with the lowest *V*. *cholerae* detection were drinking water (12.1%), shaved ice (10.3%) and street food (15.5%). Chi-squared and the Fisher’s exact test showed that there was no significant difference in the overall percentage of *V*. *cholerae* positive samples between the two neighbourhoods.

### Quantified *V*. *cholerae* positive samples

Of the 229 samples that were positive for *V*. *cholerae* (Ct values <39) in both neighbourhoods, 164 samples were quantifiable (Ct values of <36). **Table 2** shows the *V*. *cholerae* positive samples that had a Ct value of <36, therefore, *V*. *cholerae* concentration in these samples were quantified. All the surface water samples collected had Ct values of <36, therefore, all surface water samples were quantified, however, less than 50% of *V*. *cholerae* positive drinking water were quantifiable since majority of the drinking water samples had Ct values >36.

**Table 2:** Sample size and percent of quantifiable positive for *V*. *cholerae* by neighbourhood.

Percent of Positive Samples (Number of Positive/Number of Samples)			
Sample types	Mukuru Kwa Reuben	Mukuru Kwa Njenga	Total
Drinking water^†^	6.5 (7/107)	4.0 (6/150)	5.1 (13/257)
Flood water	60.0 (9/15)	80.0 (12/15)	70.0 (21/30)
Open drain	73.3 (33/45)	80.4 (37/46)	77.0 (70/91)
**Public latrine	20.0 (3/15)	26.7 (4/15)	23.3 (7/30)
Raw produce	13.3 (2/15)	20.0 (3/15)	16.7 (5/30)
Shaved ice	0 (0/14)	0 (0/15)	0 (0/29)
Soil	6.7 (1/15)	26.7 (4/15)	16.7 (5/30)
Street food*	7.1 (10/140)	3.6 (5/137)	5.4 (15/277)
Surface water	93.3 (14/15)	100 (14/14)	96.6 (28/29)
Total	20.7 (79/381)	20.1 (85/422)	20.4 (164/803)

*Street food includes french fries, githeri (mixture of beans and corn), and mandazi.

^†^Drinking water includes municipal water, borehole water, water from vendors, and stored drinking water.

** Public Latrine samples were swabs of latrine walls and door handles.

### *V*. *cholerae* concentration in positive quantifiable environmental samples

**Table 3** shows the mean *V*. *cholerae* concentration of the samples that were quantified per pathway. High concentrations of *V*. *cholerae* were detected in most of the sample types except drinking water (**Table 3**). For water samples, the highest mean concentration of *V*. *cholerae* was detected in surface water (3469 gc/mL) and the lowest concentration was observed in drinking water (25.6 gc/mL). The mean concentration of *V*. *cholerae* in the same sample type varied between the two study neighbourhoods. In Mukuru Kwa Njenga, the mean concentration of *V*. *cholerae* in surface water was approximately 5-fold higher than the concentration detected in Mukuru Kwa Reuben. Additionally, higher concentrations were also detected in drinking water, soil, and raw produce samples obtained from Mukuru kwa Njenga compared to those from Mukuru Kwa Rueben. Public latrines sampled from Mukuru Kwa Reuben had a higher mean concentration (6618 gc/swab) of *V*. *cholerae* compared to Mukuru Kwa Njenga (4811.2 gc/swab). Street food samples collected from Mukuru Kwa Reuben had a slightly higher concentration (587.6 gc/g) than in Mukuru Kwa Njenga (489 gc/g). Due to the small sample sizes of quantifiable results, a two sample t-test did not detect any significant difference in the mean concentrations of *V*. *cholerae* between two neighbourhoods.

**Table 3:** Mean *V*. *cholerae* concentration in positive quantified samples.

Sample types	Mukuru Kwa Reuben	Mukuru Kwa Njenga	Total
Drinking water^†^ gc/mL	19.4 (7.0–40.0)	32.8 (4.0–107.0)	25.6 (4.0–107.0)
Flood water gc/mL	3731.5 (43.0–27320.0)	1558.9 (25.0–7357.0)	2490.0 (25.0–27320.0)
Open drain water gc/mL	4244.4 (19.0–48730.0)	2777.4 (45.0–27665.0)	3469.0 (19.0–48730.0)
Surface water gc/mL	971.3 (38.0–3780.0)	4768.6 (150.0–19975.0)	2867.0 (38.0–19975.0)
**Public latrine gc/swab	6618.5 (4740.0–9100.4)	4811.2 (3049.7–7132.5)	5585.7 (3049.7–9100.4)
Raw produce gc/g	127.1 (69.8–184.4)	1243.1 (35.2–3553.1)	796.7 (35.2–3553.1)
Shaved ice gc/g			
Soil gc/g	309.4 (309.4–309.4)	1793.5 (79.6–5980.0)	1496.7 (79.6–5980.0)
Street food* gc/g	587.6 (125.9–1948.5)	489.0 (165.2–1021.5)	554.7 (125.9–1948.5)

*Street food includes french fries, *githeri*, and *mandazi*.

^†^Drinking water includes municipal water, borehole water, water from vendors, and stored household drinking water.

**Public Latrine samples were swabs of latrine walls and door handles.

### Temporal trend of *V*. *cholerae* detection

**Fig 1:**
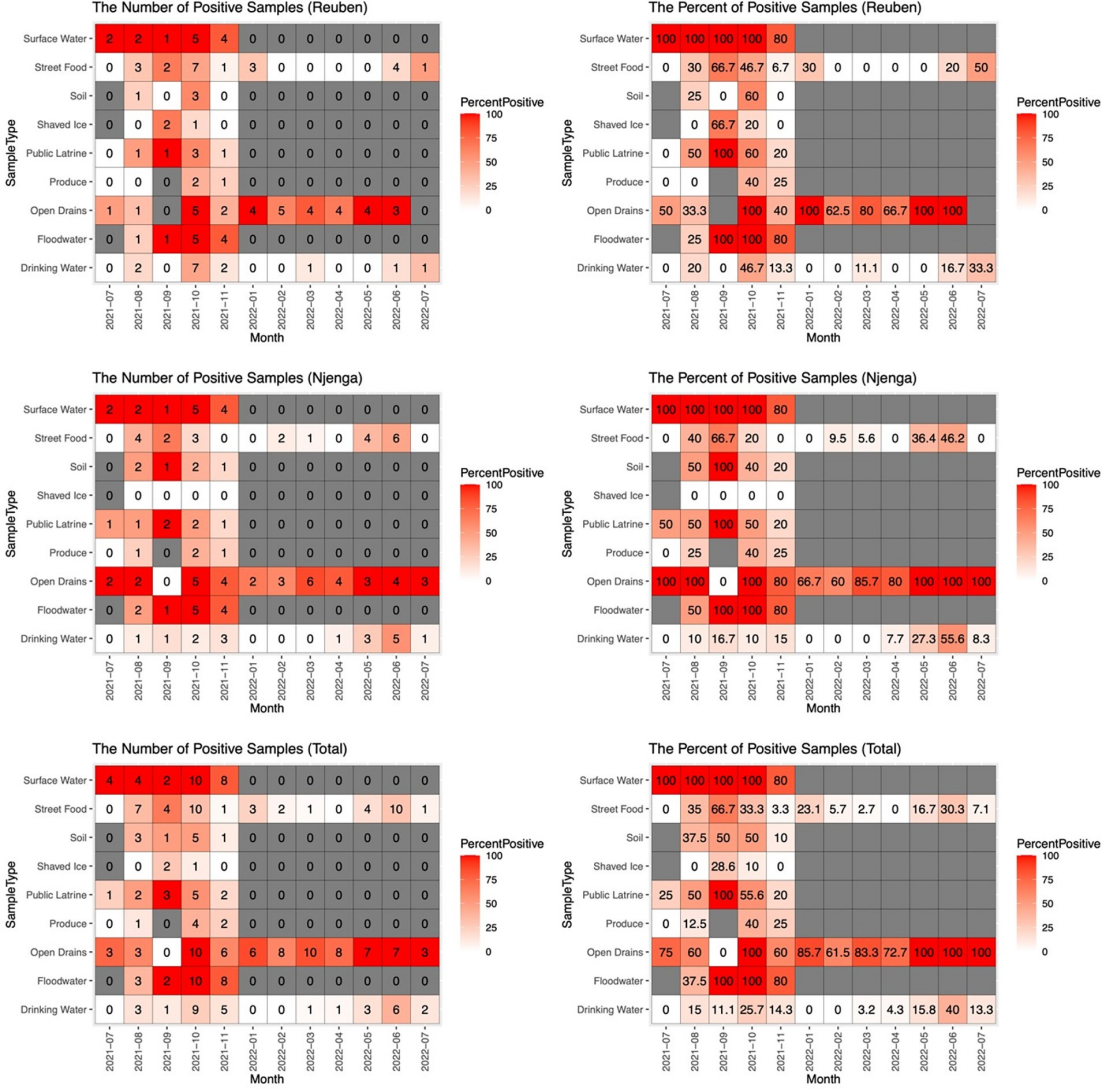
Temporal (monthly) distribution of the number of *V*. *cholerae* positive samples (left) and percentage of positive samples (right) by neighbourhood, sample type and both neighbourhoods (total), July 2021- July 2022. The color coding indicates the percent of positive samples by month in all the plots.

The detection of *V*. *cholerae* in different sample types varied between the different time periods of sampling. **Fig 1** shows the temporal distribution of the 229 *V*. *cholerae* positive samples by neighbourhood and for both neighbourhoods combined. In both neighbourhoods, *V*. *cholerae* detection in surface water and open drains remained high (≥ 50%) throughout the sampling period except in September 2021, when no positive open drain samples were detected. The percentage of positive flood water samples in both neighbourhoods (Total) was also high (≥ 80%) with the lowest detection (37.5%) observed in August. For drinking water, *V*. *cholerae* detection was slightly higher during the months of October with a gradual increase observed in the months of March through June.

### Environmental pathways with substantial contribution to total exposure to *V*. *cholerae*

**Fig 2:**
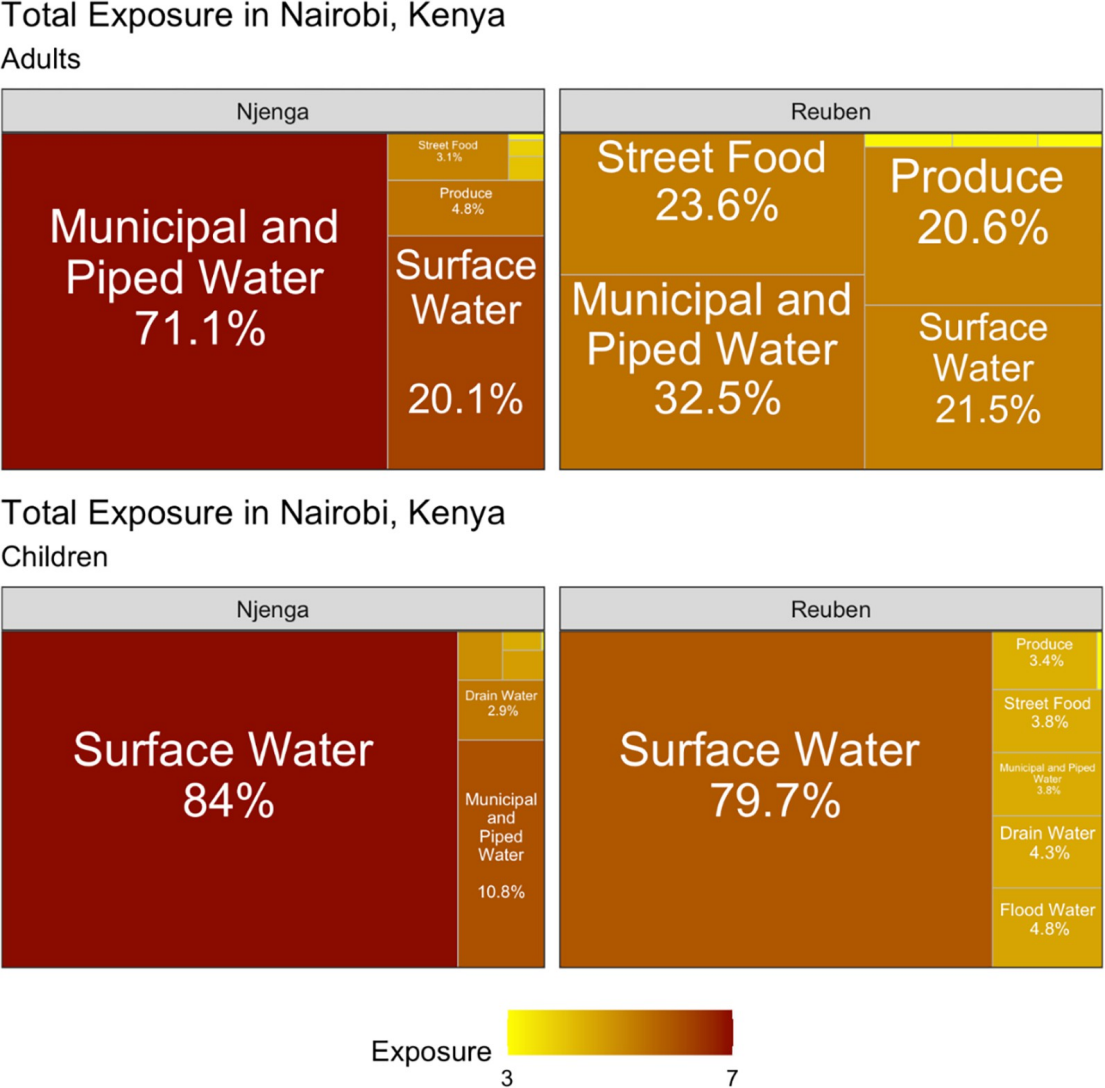
Contributions of different pathways to the total exposure to *V*. *cholerae* among adults and children in Njenga and Reuben neighbourhoods. The percentage indicates the contribution of each pathway to the total exposure. The color coding represents the magnitude of exposure to *V*. *cholerae* in every pathway.

Using the data on *V*. *cholerae* detection in environmental samples and reported behaviour frequency, we estimated exposure to *V*. *cholerae* through nine environmental pathways. The contribution of a specific pathway to the total exposure to *V*. *cholerae* was calculated by the exposure to *V*. *cholerae* from this pathway divided by the sum of the exposures to *V*. *cholerae* from all pathways. The contribution of the nine pathways to the total exposure to *V*. *cholerae* varied between adults and children. **Table 4** shows the important pathways that contributed the most to the total exposure to *V*. *cholerae*. **Fig 2** illustrates the contributions of different pathways to the total exposure of *V*. *cholerae* in both neighbourhoods and between the two age groups. Among children in both neighbourhoods, surface water was the common pathway of exposure to *V*. *cholera*e, contributing 84% and 79.7% of the total exposure to *V*. *cholerae* in Mukuru Kwa Njenga and Mukuru Kwa Reuben, respectively (**Fig 2**). Drinking water also contributed marginally (10.8%) to the total exposure to *V*. *cholerae* among children in Mukuru Kwa Njenga. Among adults, drinking water had the greatest contribution to total exposure to *V*. *cholerae*. In Mukuru Kwa Njenga, drinking water had a high (71.1%) contribution to total exposure for adults, while in Mukuru Kwa Reuben four pathways (ingestion of drinking water and street food, contact with surface water, and ingestion of raw produce) substantially contributed to the total exposure (**Fig 2**). The magnitude of the total exposure to *V*. *cholerae* varied between the two neighbourhoods and among children and adults (**Fig 2**). The total exposure to *V*. *cholerae* was higher in Mukuru kwa Njenga compared to Mukuru Kwa Reuben for both adults and children.

**Table 4:** Pathways that contributed mostto the total exposure to *V*. *cholerae* by age group and neighbourhood.

	Reuben	Njenga
**Adults**	Drinking Water, Raw Produce, Street Food, Surface Water	Drinking Water, Surface Water
**Children**	Surface Water	Drinking Water, Surface Water

## Discussion

Cholera outbreaks continue to be a major health concern in low- and middle-income countries resulting in devastating outcomes, especially among vulnerable populations such as people displaced by natural disasters (e.g., drought and flooding), in refugee camps, and those living in poor informal settlements [15, 39–42]. In Kenya, the most recent cholera outbreak was reported between October 2022 and March 2023 with a CFR of 1.6% [43]. In this study, we aimed to identify the pathways of exposure *to V*. *cholerae* and the relative contribution of each pathway to the total exposure to *V*. *cholerae* within the Mukuru informal settlement of Nairobi, which is densely populated and characterized by inadequate sanitation and hygiene.

We identified important exposure pathways to *V*. *cholerae* in the informal settlements of Nairobi. Findings from this study demonstrate the presence of *V*. *cholerae* in different environmental samples within the informal settlement, which may pose a considerable health risk to the populations residing in these areas. Although none of the environmental samples were positive for *V*. *cholerae* through culture, the hemolysin A (*hlyA*) virulence gene of *V*. *cholerae* was detected by qPCR in 20.4% of the samples. The absence of culture-positive samples in this study could imply the presence of viable but non-culturable (VBNC) or conditionally viable environmental cells (CVEC) of *V*. *cholerae* [44]. The absence of culture-positive samples could be related to both *V*. *cholerae* strains in wastewater and the laboratory methods used. *V*. *cholerae* strains surviving in wastewater with nutrient-limiting conditions may get injured, and therefore the bacteria lose their ability to produce colonies on selective media [45].These injured cells are reported to enter a state of viable but non-culturable [46]. Choosing procedures and appropriate media for the recovery of bacteria in nutrient-limited water environments is critical. A study showed that recovery of the injured bacteria on non-selective media had significantly higher numbers than on selective media [47]. Although we used an enrichment step with alkaline peptone water to try to resuscitate the *V*. *cholerae* in our environmental samples, in a future study, we may choose to also use non-selective media for recovery of *V*. *cholerae* in wastewater. Although not conducted in a slum, a study conducted in the cholera hotspots of the lake basins of Uganda reported that none of the surface water samples analysed for *V*. *cholerae* were culturable, but some samples were positive for *V*. *cholerae* genes by PCR [48]. These findings demonstrate the importance of utilizing different detection methods because dependence on culture-based methods alone may be unreliable. In the VBNC form, *V*. *cholerae* reduces metabolic activity due to environmental stress and nutrient deprivation and is not detectable through culture-based techniques [49] but remains viable for more than a year [50]. The detection of *V*. *cholerae* through qPCR in this study could be an indication of the widespread presence of VBNC in this informal settlement and underscores the need for utilizing molecular methods for the detection of *V*. *cholerae*, especially in cholera endemic areas. The presence of *V*. *cholerae* virulence genes in environmental samples in Mukuru Informal settlement is of great public health risk considering that VBNC *V*. *cholerae* cells can become infectious after ingestion by humans [51] and/or in favourable conditions [52, 53], which could result in cholera outbreaks.

In the current study, *V*. *cholerae* was detected by qPCR in > 96% of surface water samples, which was much higher than the 10.8% detection rate reported in the lake basins of Uganda [48], which is not an urban informal settlement. In Dhaka, Bangladesh, *V*. *cholerae* was detected in a large percentage of open drain samples (> 93%) from densely populated, low-income urban neighbourhoods [35], which is comparable to what was observed in this study. The high concentrations of *V*. *cholerae* in environmental waters, including surface water, open drain water, and flood water, observed in this study highlight the poor sanitation conditions in the informal settlements which suggests potential risk of adverse health effects for people who are exposed. The surface water in these areas is part of the Ngong River [54], and considering that some households are built on the river banks [55], there is a possibility that fecal waste from the households ends up in open drains and into the river. The detection of *V*. *cholerae* in the environmental samples throughout the study period may be due to the continuous poor disposal of fecal matter into the open drains and surface waters and the long-term persistence of *V*. *cholerae* in the environment.

Given that cholera outbreaks have previously been associated with contaminated drinking water [56–58], the presence of *V*. *cholerae* in drinking water and street food is worrying since it indicates possible exposure to pathogenic *V*. *cholerae* which could result in epidemics in communities that frequently buy street foods and rarely treat drinking water at home.

Although there was a difference in the contribution of different pathways to the total exposure to *V*. *cholerae* among adults, four pathways (drinking water, surface water, street food and produce) were prominent. Drinking water made a substantial contribution to the total exposure for adults, especially in Mukuru Kwa Njenga. The exposure to *V*. *cholerae* among adults through drinking water is an indication of consumption of water that is not treated or inadequately treated, which is common in the informal settlement [33]. Although the *V*. *cholerae* DNA in drinking water seems to be less prevalent and with a relatively low concentration, the frequent and large amount of direct ingestion could make it an important pathway of *V*. *cholerae* exposure. In addition, household contamination of water stored in open containers is highly likely in these households. However, it is worth noting that chlorine was detected in the sampled drinking water (**S1 Table**). The detection of *V*. *cholerae* in water containing chlorine could suggest presence of rugose (wringled) variant of *V*.*cholerae* which is thought to be resistant to chlorine [59].

For children in both neighbourhoods, surface water had the highest contribution to total exposure to *V*. *cholerae*. In addition to the high concentration of *V*. *cholerae* in surface water, children may be exposed to *V*. *cholerae* when playing around or crossing the surface water (Ngong River) through informally constructed bridges. This underscores the need for better infrastructure in the area and sensitization of residents on the impact of proper waste management. Our findings indicate that levels of exposure to *V*. *cholerae* were higher in Mukuru Kwa Njenga for both children and adults. This could be caused by a higher concentration of *V*. *cholerae* in the environment and more frequent contact with the environment in Mukuru kwa Njenga. This could be attributed to the high population density in Mukuru Kwa Njenga and the fact that the WASH infrastructure is much better in Mukuru Kwa Reuben. Our findings show that multiple pathways contribute to exposure to *V*. *cholerae* among vulnerable populations, therefore, oral cholera vaccination in the short term, and improvement of WASH infrastructure and services in the longer term are critical for the prevention and control of cholera outbreaks.

Our findings also showed that in the three sample types (drinking water, open drains, and street foods) collected for one year, *V*. *cholerae* detection peaked during two time periods; May and September–October, which are part of the rainy/wet seasons in Kenya. Similar findings were observed in Uganda, where the detection of *V*. *cholerae* peaked in the months of March–July, which corresponded to the wet seasons [48]. *V*. *cholerae* contamination in the environment may be more likely to be transferred to street food because of poor hygiene and food handling practices during months when there is more *V*. *cholerae* in the environment.

Although *V*. *cholerae* was detected in drinking water, street food, and produce no cholera outbreak was reported during the study period. This could be because the contamination levels were not sufficiently high to cause infection in most exposure incidents. However, there may be under-ascertainment and underreporting of cholera in these poor neighbourhoods as not all cases of diarrhea are reported or receive treatment at healthcare facilities. In October 2022, two months after the completion of the study, there was a cholera outbreak with a high burden of cases reported in our study sites. Low numbers of reported cholera cases (less than 20 cases) during the study period could be an indication that traditional case-based passive surveillance may not be sufficient to indicate the true circulation of *V*. *cholerae* in the population. Environmental surveillance may be useful in these settings to complement conventional case-based surveillance to monitor endemic and seasonal epidemic cholera. This is critical considering studies have reported that major cholera outbreaks are preceded by an increase in the presence of *V*. *cholerae* in the environment [49].

**In conclusion, this** study identified the probable routes of exposure to *V*. *cholerae* in an informal settlement and quantitatively assessed exposure to *V*. *cholerae* through a diverse number of environmental pathways for children and adults. Three sample types (drinking water, street food, and open drain water) samples were collected weekly over a period of 12 months to examine temporal patterns in *V*. *cholerae* detection. This study was conducted in several zones within cholera-prone informal settlements and provides insights about spatial variation in the presence of *V*. *cholerae* in the environment across small geographic areas. We also utilized both molecular and conventional methods for the detection of *V*. *cholerae* in environmental samples, and clearly attributing human behaviour to exposure to *V*. *cholerae* in the environment.

This study has several limitations. First, there were only five sampling points for every sample type per neighbourhood, which may not have been sufficient to represent the entire neighbourhood. Second, the two study neighbourhoods form a small section of a larger informal settlement, and the findings may not be generalizable to other parts of the informal settlements. Third, whereas we measured exposure, it does not directly translate to risk of infection or disease. Fourth, the hemolysin A gene detected by PCR in the environmental samples allows for the identification of virulent *V*. *cholerae* species but does not indicate if the strains are toxigenic (serogroups 01 and 0139) or NOVC. Fifth, the enrichment and culture methods used in the laboratory may not have been sufficient to effectively resuscitate VBNC *V*. *cholerae* cells.

Further studies should be undertaken to determine the phylogenetic relatedness and antimicrobial resistance in *V*. *cholerae* from both clinical and environmental sources within the informal settlement, and their role in sporadic cholera outbreaks in the informal settlement.

## Supporting information

S1 Table Number of water samples and the mean concentration of chlorine (mg/L) detected. (DOCX)

S1 Text Exposure assessment. (DOCX)

## References

[1] Yoon S hun, Waters CM. Vibrio Cholerae. Trends Microbiol [Internet]. 2019 Sep 1;27(9):806–7. Available from: doi: 10.1016/j.tim.2019.03.005 31029488 PMC6713289

[2] Cho JY, Liu R, Macbeth JC, Hsiao A. The Interface of Vibrio cholerae and the Gut Microbiome. Gut Microbes [Internet]. 2021 Jan 1;13(1):1937015. Available from: doi: 10.1080/19490976.2021.1937015 34180341 PMC8244777

[3] Nelson EJ, Harris JB, Morris JGJ, Calderwood SB, Camilli A. Cholera transmission: the host, pathogen and bacteriophage dynamic. Nat Rev Microbiol. 2009 Oct;7(10):693–702. doi: 10.1038/nrmicro2204 19756008 PMC3842031

[4] Weill FX, Domman D, Njamkepo E, Tarr C, Rauzier J, Fawal N, et al. Genomic history of the seventh pandemic of cholera in Africa. Science (80-) [Internet]. 2017 Nov 10;358(6364):785–9. Available from: doi: 10.1126/science.aad5901 29123067

[5] Smith AM, Weill FX, Njamkepo E, Ngomane HM, Ramalwa N, Sekwadi P, et al. Emergence of Vibrio cholerae O1 Sequence Type 75, South Africa, 2018–2020. Emerg Infect Dis. 2021 Nov;27(11):2927–31. doi: 10.3201/eid2711.211144 34670657 PMC8544974

[6] Mavian C, Paisie TK, Alam MT, Browne C, Beau De Rochars VM, Nembrini S, et al. Toxigenic Vibrio cholerae evolution and establishment of reservoirs in aquatic ecosystems. Proc Natl Acad Sci [Internet]. 2020 Apr 7;117(14):7897–904. Available from: doi: 10.1073/pnas.1918763117 32229557 PMC7149412

[7] Zhang Q, Alter T, Fleischmann S. Non-O1/Non-O139 Vibrio cholerae—An Underestimated Foodborne Pathogen? An Overview of Its Virulence Genes and Regulatory Systems Involved in Pathogenesis. Vol. 12, Microorganisms. 2024. 10.3390/microorganisms12040818 PMC11052320 38674762

[8] Dorman MJ, Domman D, Uddin MI, Sharmin S, Afrad MH, Begum YA, et al. High quality reference genomes for toxigenic and non-toxigenic Vibrio cholerae serogroup O139. Sci Rep [Internet]. 2019;9(1):5865. Available from: doi: 10.1038/s41598-019-41883-x 30971707 PMC6458141

[9] Octavia S, Salim A, Kurniawan J, Lam C, Leung Q, Ahsan S, et al. Population Structure and Evolution of Non-O1/Non-O139 Vibrio cholerae by Multilocus Sequence Typing. PLoS One [Internet]. 2013 Jun 11;8(6):e65342. Available from: doi: 10.1371/journal.pone.0065342 23776471 PMC3679125

[10] Davis W, Narra R, Mintz ED. Cholera. Curr Epidemiol Reports [Internet]. 2018;5(3):303–15. Available from: 10.1007/s40471-018-0162-z PMC8040429 33850688

[11] Perez-Saez J, Lessler J, Lee EC, Luquero FJ, Malembaka EB, Finger F, et al. The seasonality of cholera in sub-Saharan Africa: a statistical modelling study. Lancet Glob Heal [Internet]. 2022 Jun 1;10(6):e831–9. Available from: doi: 10.1016/S2214-109X(22)00007-9 35461521 PMC9090905

[12] Zheng Q, Luquero FJ, Ciglenecki I, Wamala JF, Abubakar A, Welo P, et al. Cholera outbreaks in sub-Saharan Africa during 2010–2019: a descriptive analysis. Int J Infect Dis [Internet]. 2022 Sep 1;122:215–21. Available from: doi: 10.1016/j.ijid.2022.05.039 35605949 PMC9439956

[13] Kiama C, Okunga E, Muange A, Marwanga D, Langat D, Kuria F, et al. Mapping of cholera hotspots in Kenya using epidemiologic and water, sanitation, and hygiene (WASH) indicators as part of Kenya’s new 2022–2030 cholera elimination plan. PLoS Negl Trop Dis. 2023 Mar;17(3):e0011166. doi: 10.1371/journal.pntd.0011166 36930650 PMC10058159

[14] NMSCEP. National multi-sectoral cholera elimination plan (nmcep) 2022–2030. 2022;2022–30. Available from: https://www.gtfcc.org/wp-content/uploads/2022/09/national-cholera-plan-kenya.pdf

[15] Kisera N, Luxemburger C, Tornieporth N, Otieno G, Inda J. A descriptive cross-sectional study of cholera at Kakuma and Kalobeyei refugee camps, Kenya in 2018. Pan Afr Med J. 2020;37:197. doi: 10.11604/pamj.2020.37.197.24798 33505566 PMC7813661

[16] Golicha Q, Shetty S, Nasiblov O, Hussein A, Wainaina E, Obonyo M, et al. Cholera Outbreak in Dadaab Refugee Camp, Kenya—November 2015-June 2016. MMWR Morb Mortal Wkly Rep. 2018 Aug;67(34):958–61. doi: 10.15585/mmwr.mm6734a4 30161101 PMC6124821

[17] Hudson Taabukk K, Waqo B, Zeinab G, George G, Robert M, Jacob R, et al. A protracted cholera outbreak among residents in an urban setting, Nairobi county, Kenya, 2015. Pan Afr Med J [Internet]. 2020;36(127). Available from: https://www.panafrican-med-journal.com/content/article/36/127/full 10.11604/pamj.2020.36.127.19786 PMC7422748 32849982

[18] Ortega DR, Kjær A, Briegel A. The chemosensory systems of Vibrio cholerae. Mol Microbiol [Internet]. 2020 Sep 1;114(3):367–76. Available from: doi: 10.1111/mmi.14520 32347610 PMC7534058

[19] Debnath A, Miyoshi S ichi. The Impact of Protease during Recovery from Viable but Non-Culturable (VBNC) State in Vibrio cholerae. Vol. 9, Microorganisms. 2021. 10.3390/microorganisms9122618 PMC8707003 34946219

[20] Ayibieke A, Nishiyama A, Senoh M, Hamabata T. Gene expression analysis during the conversion from a viable but nonculturable to culturable state in Vibrio cholerae. Gene [Internet]. 2023;863:147289. Available from: https://www.sciencedirect.com/science/article/pii/S0378111923001300 doi: 10.1016/j.gene.2023.147289 36804851

[21] Shuo Z, Xin L, JingYun Z, Biao K. Absolute Quantification of Viable but Nonculturable Vibrio cholerae Using Droplet Digital PCR with Oil-Enveloped Bacterial Cells. Microbiol Spectr [Internet]. 2022 Jun 28;10(4):e00704–22. Available from: doi: 10.1128/spectrum.00704-22 35762749 PMC9430983

[22] Fernández-Delgado M, García-Amado MA, Contreras M, Incani RN, Chirinos H, Rojas H, et al. Survival, induction and resuscitation of Vibrio cholerae from the viable but non-culturable state in the Southern Caribbean Sea. Rev Inst Med Trop Sao Paulo. 2015;57(1):21–6. doi: 10.1590/S0036-46652015000100003 25651322 PMC4325519

[23] WHO/Department of Control of Neglected Tropical Diseases. Weekly epidemiological record Relevé épidémiologique hebdomadaire. Wkly Epidemiol Rec [Internet]. 2018;16(93):201–20. Available from: http://www.who.int/wer2018,93,649-660No48%0Ahttp://www.who.int/wer

[24] Daboul J, Weghorst L, DeAngelis C, Plecha SC, Saul-McBeth J, Matson JS. Characterization of Vibrio cholerae isolates from freshwater sources in northwest Ohio. PLoS One [Internet]. 2020 Sep 3;15(9):e0238438. Available from: doi: 10.1371/journal.pone.0238438 32881972 PMC7470319

[25] Zohra T, Ikram A, Salman M, Amir A, Saeed A, Ashraf Z, et al. Wastewater based environmental surveillance of toxigenic Vibrio cholerae in Pakistan. PLoS One [Internet]. 2021 Sep 30;16(9):e0257414. Available from: doi: 10.1371/journal.pone.0257414 34591885 PMC8483414

[26] Jutla A, Whitcombe E, Hasan N, Haley B, Akanda A, Huq A, et al. Environmental factors influencing epidemic cholera. Am J Trop Med Hyg. 2013 Sep;89(3):597–607. doi: 10.4269/ajtmh.12-0721 23897993 PMC3771306

[27] Jones FK, Wamala JF, Rumunu J, Mawien PN, Kol MT, Wohl S, et al. Successive epidemic waves of cholera in South Sudan between 2014 and 2017: a descriptive epidemiological study. Lancet Planet Heal. 2020 Dec;4(12):e577–87. doi: 10.1016/S2542-5196(20)30255-2 33278375 PMC7750463

[28] Ren H, Guo W, Zhang Z, Kisovi LM, Das P. Population density and spatial patterns of informal settlements in Nairobi, Kenya. Sustain. 2020;12(18).

[29] UN-Habitat. Kenya COUNTRY Brief. 2023; Available from: https://unhabitat.org/sites/default/files/2023/07/kenya_country_brief_final_en.pdf. (Accessed on 17 November 2023)

[30] Robb K, Null C, Teunis P, Yakubu H, Armah G, Moe CL. Assessment of Fecal Exposure Pathways in Low-Income Urban Neighborhoods in Accra, Ghana: Rationale, Design, Methods, and Key Findings of the SaniPath Study. Am J Trop Med Hyg. 2017 Oct;97(4):1020–32. doi: 10.4269/ajtmh.16-0508 28722599 PMC5637580

[31] Wang Y, Moe CL, Null C, Raj SJ, Baker KK, Robb KA, et al. Multipathway Quantitative Assessment of Exposure to Fecal Contamination for Young Children in Low-Income Urban Environments in Accra, Ghana: The SaniPath Analytical Approach. Am J Trop Med Hyg. 2017 Oct;97(4):1009–19. doi: 10.4269/ajtmh.16-0408 29031283 PMC5637579

[32] Raj SJ, Wang Y, Yakubu H, Robb K, Siesel C, Green J, et al. The SaniPath Exposure Assessment Tool: A quantitative approach for assessing exposure to fecal contamination through multiple pathways in low resource urban settlements. PLoS One. 2020;15(6):e0234364. doi: 10.1371/journal.pone.0234364 32530933 PMC7292388

[33] Corburn J, Njoroge P, Weru J, Musya M. Urban Climate Justice, Human Health, and Citizen Science in Nairobi’s Informal Settlements. Vol. 6, Urban Science. 2022.

[34] Rojas A, Nomedji K, West CT. Walking the Line: Conducting Transect Walks in Burkina Faso. Pract Anthropol. 2021;43(1):18–21. doi: 10.17730/0888-4552.43.1.18 34720394 PMC8550581

[35] Amin N, Rahman M, Raj S, Ali S, Green J, Das S, et al. Quantitative assessment of fecal contamination in multiple environmental sample types in urban communities in Dhaka, Bangladesh using SaniPath microbial approach. PLoS One [Internet]. 2019 Dec 16;14(12):e0221193. Available from: doi: 10.1371/journal.pone.0221193 31841549 PMC6913925

[36] Wang Y, Mairinger W, Raj SJ, Yakubu H, Siesel C, Green J, et al. Quantitative assessment of exposure to fecal contamination in urban environment across nine cities in low-income and lower-middle-income countries and a city in the United States. Sci Total Environ. 2022 Feb;806(Pt 3):151273. doi: 10.1016/j.scitotenv.2021.151273 34718001 PMC8651627

[37] Jianwei H, Yumei Z, Huixin W, Jiafeng Z, Shijie H, Jianjun N, et al. Quadruplex Real-Time PCR Assay for Detection and Identification of Vibrio cholerae O1 and O139 Strains and Determination of Their Toxigenic Potential. Appl Environ Microbiol [Internet]. 2009 Nov 15;75(22):6981–5. Available from: doi: 10.1128/AEM.00517-09 19767462 PMC2786538

[38] Amin N, Liu P, Foster T, Rahman M, Miah MR, Ahmed GB, et al. Pathogen flows from on-site sanitation systems in low-income urban neighborhoods, Dhaka: A quantitative environmental assessment. Int J Hyg Environ Health [Internet]. 2020;230:113619. Available from: https://www.sciencedirect.com/science/article/pii/S1438463920305654 doi: 10.1016/j.ijheh.2020.113619 32942223

[39] Shannon K, Hast M, Azman AS, Legros D, McKay H, Lessler J. Cholera prevention and control in refugee settings: Successes and continued challenges. PLoS Negl Trop Dis. 2019 Jun;13(6):e0007347. doi: 10.1371/journal.pntd.0007347 31220084 PMC6586254

[40] Jain A, Choudhary S, Saroha E, Bhatnagar P, Harvey P. Cholera Outbreak in an Informal Settlement at Shahpur Huts, Panchkula District, Haryana State, India, 2019. Indian J Public Health [Internet]. 2021;65(5). Available from: https://journals.lww.com/IJPH/Fulltext/2021/65001/Cholera_Outbreak_in_an_Informal_Settlement_at.11.aspx 10.4103/ijph.IJPH_970_20 PMC10467578 33753593

[41] Simpson RB, Babool S, Tarnas MC, Kaminski PM, Hartwick MA, Naumova EN. Dynamic mapping of cholera outbreak during the Yemeni Civil War, 2016–2019. J Public Health Policy [Internet]. 2022;43(2):185–202. Available from: doi: 10.1057/s41271-022-00345-x 35614203 PMC9192410

[42] Piarroux R, Moore S, Rebaudet S. Cholera in Haiti. Presse Med [Internet]. 2022;51(3):104136. Available from: https://www.sciencedirect.com/science/article/pii/S075549822200029X doi: 10.1016/j.lpm.2022.104136 35705115

[43] Africa WHORO for. Weekly Bulletin on Outbreak and other Emergencies:Week 17: 17–23 April 2023. Available from: https://apps.who.int/iris/handle/10665/367270

[44] Conner JG, Teschler JK, Jones CJ, Yildiz FH. Staying Alive: Vibrio cholerae’s Cycle of Environmental Survival, Transmission, and Dissemination. Microbiol Spectr. 2016 Apr;4(2). doi: 10.1128/microbiolspec.VMBF-0015-2015 27227302 PMC4888910

[45] Hoadley AW, Cheng CM. The Recovery of Indicator Bacteria on Selective Media. J Appl Bacteriol [Internet]. 1974 Mar 1;37(1):45–57. Available from: doi: 10.1111/j.1365-2672.1974.tb00413.x 4211091

[46] Chmielewski RAN, Frank JF. Formation of viable but nonculturable Salmonella during starvation in chemically defined solutions. Lett Appl Microbiol [Internet]. 1995 Jun 1;20(6):380–4. Available from: doi: 10.1111/j.1472-765x.1995.tb01326.x 7786506

[47] Rajkowski KT, Rice EW. Recovery and survival of Escherichia coli O157:H7 in reconditioned pork-processing wastewater. J Food Prot [Internet]. 1999;62(7):731–4. Available from: doi: 10.4315/0362-028x-62.7.731 10419263

[48] Bwire G, Debes AK, Orach CG, Kagirita A, Ram M, Komakech H, et al. Environmental Surveillance of Vibrio cholerae O1/O139 in the Five African Great Lakes and Other Major Surface Water Sources in Uganda [Internet]. Vol. 9, Frontiers in Microbiology. 2018. Available from: https://www.frontiersin.org/articles/10.3389/fmicb.2018.01560 10.3389/fmicb.2018.01560 PMC6085420 30123189

[49] Islam MS, Zaman MH, Islam MS, Ahmed N, Clemens JD. Environmental reservoirs of Vibrio cholerae. 2020;38:52–62. 10.1016/j.vaccine.2019.06.033 31285087

[50] Islam MS, Islam MS, Mahmud ZH, Cairncross S, Clemens JD, Collins AE. Role of phytoplankton in maintaining endemicity and seasonality of cholera in Bangladesh. Trans R Soc Trop Med Hyg [Internet]. 2015 Sep 1;109(9):572–8. Available from: doi: 10.1093/trstmh/trv057 26179653

[51] Colwell RR, Brayton P, Herrington D, Tall B, Huq A, Levine MM. Viable but non-culturable Vibrio cholerae O1 revert to a cultivable state in the human intestine. World J Microbiol Biotechnol. 1996;12:28–31. doi: 10.1007/BF00327795 24415083

[52] Brenzinger S, van der Aart LT, van Wezel GP, Lacroix JM, Glatter T, Briegel A. Structural and Proteomic Changes in Viable but Non-culturable Vibrio cholerae. Front Microbiol. 2019;10:793. doi: 10.3389/fmicb.2019.00793 31057510 PMC6479200

[53] Senoh M, Ghosh-Banerjee J, Ramamurthy T, Hamabata T, Kurakawa T, Takeda M, et al. Conversion of viable but nonculturable Vibrio cholerae to the culturable state by co-culture with eukaryotic cells. Microbiol Immunol [Internet]. 2010 Sep 1;54(9):502–7. Available from: doi: 10.1111/j.1348-0421.2010.00245.x 20840148

[54] Vane CH, Kim AW, Lopes dos Santos RA, Gill JC, Moss-Hayes V, Mulu JK, et al. Impact of organic pollutants from urban slum informal settlements on sustainable development goals and river sediment quality, Nairobi, Kenya, Africa. Appl Geochemistry [Internet]. 2022;146:105468. Available from: https://www.sciencedirect.com/science/article/pii/S0883292722002724

[55] Greibe Andersen J, Kallestrup P, Karekezi C, Yonga G, Kraef C. Climate change and health risks in Mukuru informal settlement in Nairobi, Kenya–knowledge, attitudes and practices among residents. BMC Public Health [Internet]. 2023;23(1):393. Available from: doi: 10.1186/s12889-023-15281-y 36841782 PMC9958313

[56] Eurien D, Mirembe BB, Musewa A, Kisaakye E, Kwesiga B, Ogole F, et al. Cholera outbreak caused by drinking unprotected well water contaminated with faeces from an open storm water drainage: Kampala City, Uganda, January 2019. BMC Infect Dis [Internet]. 2021;21(1):1281. Available from: doi: 10.1186/s12879-021-07011-9 34961483 PMC8711146

[57] Kwesiga B, Pande G, Ario AR, Tumwesigye NM, Matovu JKB, Zhu BP. A prolonged, community-wide cholera outbreak associated with drinking water contaminated by sewage in Kasese District, western Uganda. BMC Public Health [Internet]. 2017;18(1):30. Available from: doi: 10.1186/s12889-017-4589-9 28720083 PMC5516304

[58] Okello PE, Bulage L, Riolexus AA, Kadobera D, Kwesiga B, Kajumbula H, et al. A cholera outbreak caused by drinking contaminated river water, Bulambuli District, Eastern Uganda, March 2016. BMC Infect Dis. 2019 Jun;19(1):516. doi: 10.1186/s12879-019-4036-x 31185939 PMC6558808

[59] Morris JG Jr, Sztein MB, Rice EW, Nataro JP, Losonsky GA, Panigrahi P, et al. Vibrio cholerae 01 can assume a chlorine-resistant rugose survival form that is virulent for humans. J Infect Dis. 1996;174(6):1364–8. 8940236 10.1093/infdis/174.6.1364

