# Supplementary material for: Pathways of exposure to Vibrio Cholerae in an urban informal settlement in Nairobi, Kenya
Source: PLOS Glob Public Health. 2024 Aug 20;4(8):e0002880. doi: 10.1371/journal.pgph.0002880 (PMC11335117; doi:10.1371/journal.pgph.0002880)
Supplement: S1 Table — (DOCX) [file pgph.0002880.s001.docx]

Number of water samples and the mean (range) concentration of chlorine (mg/L) detected.

|  | Water Type | | | |
| --- | --- | --- | --- | --- |
|  | Municipal Drinking Water | Water Vendor Drinking Water | Borehole Drinking Water | Stored Water |
| No of samples | 91 | 45 | 91 | 8 |
| No of samples with detectable chlorine | 91 | 45 | 87 | 8 |
| Free chlorine (mg/L) | 1.35 (0.36–2.63) | 1.12 (0.40–2.02) | 0.93 (0.22–1.76) | 1.15 (0.37–1.94) |
| Total chlorine (mg/L) | 1.98 (0.48–2.95) | 1.82 (0.34–3.06) | 1.38 (0–2.42) | 1.85 (0.68–2.73) |
