# Supplementary material for: Pathways of exposure to Vibrio Cholerae in an urban informal settlement in Nairobi, Kenya
Source: PLOS Glob Public Health. 2024 Aug 20;4(8):e0002880. doi: 10.1371/journal.pgph.0002880 (PMC11335117; doi:10.1371/journal.pgph.0002880)
Supplement: S1 Text — (DOCX) [file pgph.0002880.s002.docx]

**Exposure Assessment**

The concentration of *V. cholerae* in samples from a specific type, $sp$, at neighbourhood $nb$ is modelled as ${conc}_{sp,nb}\sim lognormal(\mu_{sp,nb}, \sigma_{sp,nb}^{2})$. For each sample type, the concentrations of non-quantifiable samples were substituted with the sample type specific lower limit of quantification, which is the minimum of concentrations from quantifiable samples in this sample type. The frequency of contact behaviour that lead to exposure through pathway $pw$ for a specific $age$ group (adults vs. children) at neighbourhood $nb$ is modelled as a negative binomial distribution, ${freq}_{pw, age,nb}\sim NB(r_{pw, age,nb},p_{pw, age,nb})$. The Bayesian analyses were conducted in JAGS and the posterior means were used as the Bayes estimators of parameters.

For each pathway, neighbourhood, and age group combination, 1000 Monte Carlo simulations were conducted by drawing samples from the distributions of *V. cholerae* concentration and contact frequency with estimated parameters. In each simulation, the exposure was calculated by $E_{pw, age,nb}={conc}_{sp,nb}\times{freq}_{pw, age,nb}\times{intake}_{pw,age}$ based on pathway, age group, and neighbourhood. ${intake}_{pw,age}$ is the intake volume per contact, which can be found in the Appendix S2 of Raj et al. (2020). The simulations generate estimates of exposure to *V. cholerae* through a specific exposure pathway for adults or children in a specific neighbourhood.
